# Monitoring Athletes during Training Camps: Observations and Translatable Strategies from Elite Road Cyclists and Swimmers

**DOI:** 10.3390/sports6030063

**Published:** 2018-07-20

**Authors:** Anna E. Saw, Shona L. Halson, Iñigo Mujika

**Affiliations:** 1 Cricket Australia, 60 Jolimont St, Jolimont, VIC 3002, Australia; 2 AIS Physiology, Australian Institute of Sport, Leverrier St, Bruce, ACT 2617, Australia; shona.halson@gmail.com; 3 Department of Physiology, Faculty of Medicine and Odontology, University of the Basque Country, 48940 Leioa, Basque Country, Spain; inigo.mujika@inigomujika.com; 4 Exercise Science Laboratory, School of Kinesiology, Faculty of Medicine, Universidad Finis Terrae, Santiago 1509, Chile

**Keywords:** altitude, body composition, sleep, heart rate, hydration

## Abstract

Monitoring is an essential yet unstandardized component of managing athletic preparation. The purpose of this paper is to provide insight into the typical measurements and responses observed from monitoring elite road cyclist and swimmers during training camps, and translate these observations to practical strategies for other practitioners to employ. Twenty-nine male professional cyclists, 12 male and 19 female international swimmers participated in up to three of the eight 4–19 day training camps, held early in the season or leading into major competitions, at sea-level or moderate altitude. Monitoring included body mass and composition, subjective sleep, urinary specific gravity (USG), resting heart rate (HR) and peripheral oxygen saturation (SpO_2_) at altitude. Sum of seven skinfolds most likely decreased in the order of 3.1 ± 3.6 mm week-to-week, accompanied by a most likely trivial decrease in body mass of 0.4 ± 0.4 kg week-to-week. At altitude, sleep quality very likely trivially improved week-to-week (0.3 ± 0.3 AU), SpO_2_ possibly increased week-to-week (0.6 ± 1.7%), whilst changes in resting HR were unclear (0 ± 4 bpm). Sleep duration and USG were stable. Comparing individual to group day-to-day change in monitored variables may prove effective to flag athletes potentially at risk of training maladaptation. Practitioners may replicate these methods to establish thresholds specific to their cohort and setting. This study provides further support for a multi-faceted approach to monitoring elite athletes in training camp environments.

## 1. Introduction

Elite athletic preparation requires a fine balance between pushing the training and adaptation boundaries for performance, and avoiding negative outcomes such as underperformance, injury, or poor wellbeing [1,2]. Monitoring is therefore considered an essential component of managing athletic preparation [3]. Monitoring efforts may be directed towards: (a) Ensuring athletes are healthy and therefore receptive to positive training adaptations; (b) quantifying the physiological stress experienced; and (c) assessing the physiological and psychological response to training [1,2,3]. Despite growing research in this space, much of the data on elite athletes remains unpublished, thus hindering advancement in the field [3].

Elite athletes often participate in training camps to enhance training adaptation at specific times in the season. The nature of training camps is manipulated to align with certain goals, for instance improving aerobic or anaerobic capacities, preparing for a specific competition, and team cohesion. Training camps pose additional, often intentional, challenges to the athlete state. For instance, overreaching has been shown to be elicited with seven days of intensified training in trained cyclists [4], yet may be detectable after only three days with daily monitoring [5]. Training at altitude adds an additional dimension of complexity, with individual responses to the environmental stress of hypoxia contributing to the training response [6]. The brief (i.e., several days to several weeks) and semi-controlled structure of training camps presents an ideal setting to intensify monitoring efforts to both manage adaptation and better understand how athletes respond to training [7]. Given the complexity of athletic preparation, several physical, physiological, and psychosocial measures are typically employed to help monitor and manage elite athletes [4,5,8]. The utility of each measure is dependent upon its ability to discern between what is normal fluctuation in a measure, and what is a meaningful change requiring action. Therefore, it is important to understand how such measures typically respond in different training camp scenarios, and what thresholds may be most applicable. This is particularly important in elite athlete cohorts where very small changes can impact performance [9]. As such, there is value in reporting ‘real world’ monitoring data from elite athletes to assist practitioners working with similar cohorts and settings in the future. This could improve the use of monitoring methods by providing evidence for what constitutes a ‘normal’ response, and what thresholds may be applied to detect meaningful change.

The purpose of this study is firstly to describe the typical measurements and responses observed from monitoring elite road cyclists and swimmers during eight training camps. Camps occurred at different times of the season, and at sea-level and moderate altitude. A secondary purpose is to translate these observations to practical strategies for practitioners seeking to effectively utilize monitoring strategies with elite athletes in training camp settings.

## 2. Materials and Methods

### 2.1. Subjects and Training Camps

#### 2.1.1. Cyclists

Twenty-nine male cyclists (age 29 ± 4 years) participated in one (n = 17), two (n = 9), or three (n = 3) of the four team training camps held across a season. All cyclists were members of a UCI World Tour cycling team. The sample included an Olympic Champion and Grand Tour (Giro d’Italia, Tour de France, Vuelta a España) general classification podium finisher, and six Grand Tour stage winners. Based on the team manager’s description and according to their main role in competition, 12 cyclists were all terrain riders, 10 climbers, 5 sprinters, 1 flat terrain rider and 1 time trial specialist [10].

Four 9–12-day team training camps were conducted during the 2013 season: (i) pre-Tour Down Under (TDU) at sea-level (December 2012); (ii) early-season at sea-level (January 2013); (iii) pre-Volta a Catalunya (Volta) at sea-level (March 2013); and (iv) pre-Tour de France (TDF) at 2320 m altitude (May 2013). The main purpose of the early season camp was to set fitness foundations for the riders and work on team cohesion, whereas the other camps targeted specific competitive fitness for riders who would take part in each particular race.

#### 2.1.2. Swimmers

Thirty-one swimmers (19 female, age 21 ± 4 years; 12 male, age 23 ± 4 years) participated in one (n = 22) or two (n = 9) of the four team training camps held between 2012 and 2016. All swimmers were members of the French and Spanish pool or open water national teams, and participated in major international competitions. The sample included two Olympic champions, seven world champions, and three world record holders.

Four 4–19-day team training camps were conducted during the 2012–2016 seasons: (i) Early-season at 1360 m altitude (January 2012); (ii) pre-London Olympic Games at sea-level (July 2012); (iii) pre-Kazan World Championships at 2320 m altitude (June 2015); and (iv) pre-Rio Olympic Games at 2320 m altitude (May 2016). The main purpose of the early season camp was to set fitness foundations for the swimmers through moderate altitude after a brief 3–7 day end-of-year break, whereas the other camps targeted specific competitive fitness for swimmers who would take part in each particular competition.

### 2.2. Methods

Procedures formed part of the teams’ service provision, which conforms to the Code of Ethics of the World Medical Association (Declaration of Helsinki). Athletes provided informed consent to participate in monitoring procedures associated with team duties, with the understanding that data may be used for research purposes.

Urinary specific gravity (USG, UG-α Digital Refractometer, Atago Co., Tokyo, Japan) was monitored daily on waking, in a mid-stream urine sample. Body mass (Seca 877, Hamburg, Germany) was then monitored after voiding. Skinfolds were measured by the same accredited anthropometrist using a standard protocol for the sum of 7 sites (triceps, biceps, subscapular, supraspinale, abdominal, front thigh, medial calf; Holtain, Crymych, UK) at the start of each camp, and repeated mid-camp (pre-TDF) and at the end of the camp (pre-TDU, pre-Volta, pre-TDF). Sleep quality was subjectively rated each morning using a Likert scale (5 = very good; 4 = good; 3 = average; 2 = bad; 1 = very bad).

For camps at altitude, additional measures on waking included sleep hours (self-reported), resting heart rate (HR) and peripheral oxygen saturation (SpO_2_) (CMS50H Pulse Oximeter, Contec Medical Systems Co., Ltd., Qinhuangdao, China).

No athletes showed signs of underperformance, reduced wellness or illness, suggesting athletes remained healthy and in a functional training state throughout each camp [11]. This may be attributable to appropriate planning and day-to-day evaluation and adaptation of training prescription at an individual level, hence prescribed group training loads are not reported here.

### 2.3. Statistical Analysis

Raw data and calculated daily/weekly intra-individual changes satisfied visual inspection and the Shapiro–Wilk test for normality, with the exception of changes in body mass and skinfolds which were slightly negatively skewed (SPSS version 24, IBM, Armonk, NY, USA). Normality was not improved by transformation, hence raw body mass and skinfold data were used in analyses and mean and standard deviation (SD) were deemed acceptable to describe the centrality and dispersion of data. The practical importance of changes were calculated using magnitude based inferences [12].

The data were used to retrospectively evaluate the utility of commonly cited thresholds for daily monitoring. The smallest worthwhile change (SWC) (0.3 × SD/Mean × 100) [13] and the smallest real change (SRC) (1.96 × $\sqrt{2}$ × SD/$\sqrt{n}$, where n is the number of athletes from which the mean and SD were derived) [14] were calculated from day 1 values, and applied to all subsequent days. The z-score ((value-Mean)/SD) was calculated on a daily basis, comparing the intra-individual change to the mean group change for that day, with thresholds of 1.0 and 1.5 applied. The percentage of athletes ‘flagged’ by each criteria throughout each camp was calculated and averaged across camps.

## 3. Results

The measurements of cyclists and swimmers entering each training camp are detailed in Table 1 and Table 2, respectively. Across all camps, athletes slept on average 8.0 ± 0.5 h on the first night, and rated their sleep quality as 3.6 ± 0.8 out of 5 (average-good). Sleep was poorest for swimmers participating in the Pre-Rio camp at altitude (7.5 ± 0.3 h, quality 2.3 ± 0.7 (bad)).

**Figure 1:**
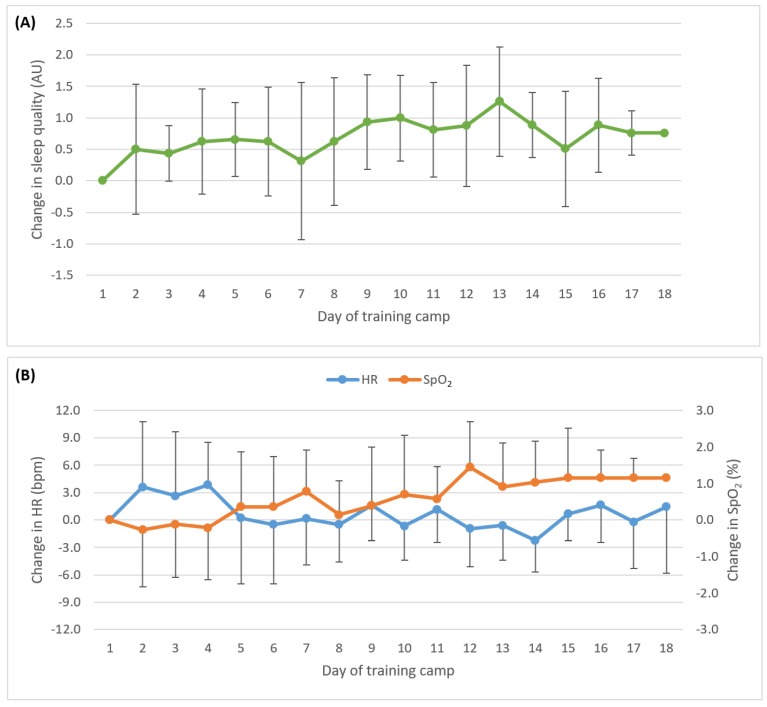
Change in sleep quality (**A**); resting heart rate and resting peripheral oxygen saturation (**B**) relative to Day 1 over the course of a training camp at altitude. Data presented as mean ± SD. Symmetrical error bars above or below the mean values have been omitted for clarity.

At altitude, the sleep quality of athletes very likely trivially improved week-to-week (mean individual weekly change 0.3 ± 0.3 AU) (Figure 1A). Peripheral oxygen saturation possibly increased week-to-week (0.6 ± 1.7%), whilst changes in resting HR were unclear (0 ± 4 bpm) (Figure 1B).

**Figure 2:**
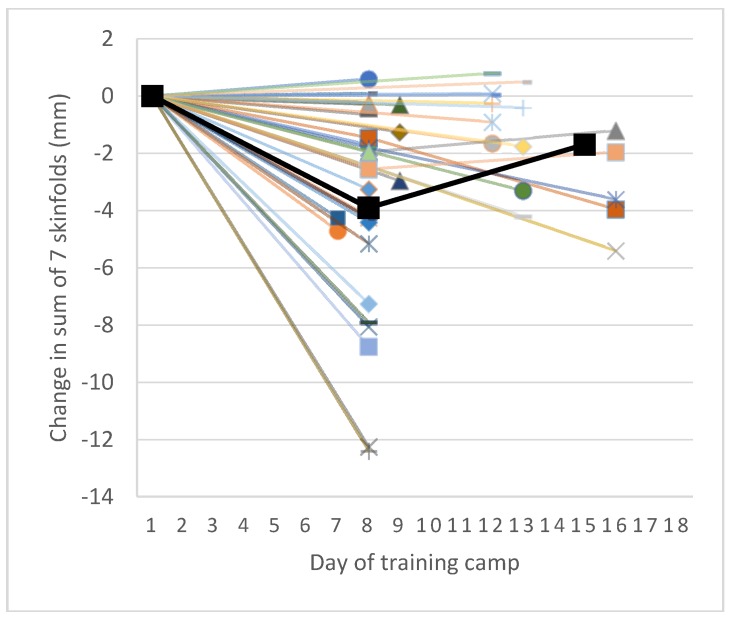
Change in sum of 7 skinfold measurements relative to Day 1 over the course of a training camp. Bold line is mean weekly change, summarizing illustrated individual responses for cyclists and swimmers, males and females, training at sea-level or altitude.

The sum of 7 skinfolds most likely decreased during training camps, with an average reduction of 3.1 ± 3.6 mm week-to-week (Figure 2). This was accompanied by a most likely trivial decrease in body mass of 0.4 ± 0.4 kg week-to-week.

When compared to the group values on day 1, 19 ± 25% of athletes exceeded the SWC threshold, and 31 ± 21% of athletes exceeded the SRC threshold (Table 3). When compared to the daily change of the group, 34 ± 3% and 16 ± 3% of athletes exceeded a z-score of 1.0 and 1.5 respectively.

## 4. Discussion

Training camp measurements of elite cyclists and swimmers have been retrospectively analysed to offer insight into what is most often privileged information. Typical responses to training camps included a shift in body composition and cardiovascular adaptation to altitude, whilst other measurements remained stable.

### 4.1. Change in Body Composition

The primary physiological change observed in elite road cyclists and swimmers during training camps was a decrease in body fat percentage as reflected by the sum of seven skinfolds. This was not necessarily accompanied by a decrease in body mass. Reductions in body fat were likely intentional and in line with individual performance goals (e.g., returning to ‘training weight’ at early season camps, or achieving ‘race weight’ at pre-competition camps). Reduced fat mass is associated with improved performance in swimmers [15,16] and cyclists who benefit from an increased power-to-weight ratio [10,17].

### 4.2. Changes Specific to Altitude

A typical pattern of cardiovascular response to altitude hypoxia was observed [18], whereby SpO_2_ was initially reduced and possibly normalized over the first week as the athletes adapted. To accommodate the reduced SpO_2_, resting HR would have been higher than normal for these athletes [18], however it was unclear whether HR decreased with time. This contrasts with the response observed in elite distance runners who experienced a reduction in HR without a clear change in SpO_2_ [8]. To more accurately ascertain the HR response, and differentiate between cardiovascular adaptations to hypoxia, improvements in fitness, or possible training maladaptation, it is recommended that athletes record their resting HR in the week prior to attending an altitude training camp.

An additional cardiovascular response to altitude hypoxia to consider is an increased blood perfusion of skeletal muscles [19]. Increased perfusion of skeletal muscles has been associated with increased muscle mass and decreased fat mass. This shift in body composition is consistent with the observed reduction in skinfolds without a concomitant decrease in body mass. Energy balance may also be shifted by reduced energy intake rather than an increased energy cost of training [20]. Athletes should therefore tailor their energy and nutrient intakes to either exploit or counter these responses to altitude, in line with their body composition goals.

The physiological stress of living at altitude is typically associated with dehydration (increased USG) [21] and poor sleep quality [22]; however, these negative responses were not observed, except for occasionally altered individual values. This likely reflects the elite athletes’ increased awareness of hydration needs and also their experience in sleeping in different environments. It is possible that sleep quality was overrated, as perceived adequate sleep duration (consistently ~8 h) may have impaired athletes’ ability to appropriately determine their sleep quality [23]. Anecdotally, athletes often report improved sleep with additional time spent at altitude, suggesting an adaptive response to the altitude. Actigraphy may be useful to provide a more objective measurement of sleep quality through an assessment of sleep efficiency.

### 4.3. Informing Training Management

Collectively, the observed physiological responses to training camps reflect the fitness and experience of the elite cohort. Subjectively, the performance of athletes whilst training at the camps and in subsequent major competitions, where applicable, suggested that the athletes responded appropriately to a well-designed and managed training program, however we acknowledge this is speculative without objective performance data during the camps. The routine monitoring of body mass, skinfolds, sleep duration and quality, USG, resting HR, and SpO_2_ may have contributed to the management of these athletes by providing additional insight to practitioners.

To facilitate the detection of possible maladaptation, practitioners may pre-set thresholds to ‘flag’ meaningful changes in measures. There is a need to balance the sensitivity and specificity of thresholds, whereby it is feasible to follow up with the number of flagged athletes. In the training camp scenario with limited numbers of athletes completing similar training, the z-score approach was most successful to identify a small number of athletes each day whose response deviated from the mean group response. Z-score thresholds of 1.0 and 1.5 remain arbitrary, and may be further refined to suit the measure and/or training camp scenario. Nevertheless, thresholds are only intended to assist the practitioner, and informal monitoring through conversations and observation of the athlete remains a large component of athlete management.

### 4.4. Practical Applications

Practitioners seeking empirically supported guidance for elite athlete monitoring practices, particularly during intense periods such as training camps, may draw upon the results, methods, or overall learnings presented in this report. Firstly, the SWC and SRC values presented in Table 1 and Table 2 may be used as thresholds for detecting meaningful change in similar cohorts and settings. However, practitioners are also required to use their judgment in the instance that these calculated values are non-sensical.

Practitioners working with different cohorts or settings, or where monitoring is already established, may use the methods outlined here as a template to establish their thresholds. With sufficient data, individual-specific parameters may also be determined. This approach may be further improved by conducting repeat measurements to determine the reliability of measures [24].

Researchers agree that to-date, there is no single measure which can be used in isolation for athlete monitoring [2]. Of greatest utility is subjective wellbeing, which may be supplemented by other subjective and objective measures [25]. This includes measures to monitor the training response, in addition to quantifying the internal and external loads. The challenge for practitioners is to weigh the evidence for measures alongside the practical limitations of their particular setting (e.g., minimizing burden for athletes and staff, cost of equipment).

In the present study, the utility of body composition, sleep, and USG for detecting possible signs of maladaptation is unclear. However, it is worth considering their potential value for raising athlete awareness and accountability of these factors which impact performance [26]. For instance, a perceived reduction in sleep duration or quality may prompt an athlete to take a nap, or a higher USG reading may prompt an athlete to drink more water. In these instances, understanding what is a meaningful change may alleviate unnecessary obsessive behaviors.

## 5. Conclusions

Elite athletes experienced shifts in body composition during training camps, likely in line with individual performance goals and possibly aided by altitude. The stability of other monitored measures suggests athletes managed themselves appropriately, with the assistance of coaching and sport science support. Comparing individual to group day-to-day change in monitored variables may prove effective to flag athletes potentially at risk of training maladaptation. Practitioners may replicate these methods to establish thresholds specific to their cohort and setting. This study provides further support for a multi-faceted approach to monitoring elite athletes in training camp environments.

## Figures and Tables

**Table 1:** Measurements of elite cyclists on day 1 of training camps, smallest worthwhile change (SWC) and smallest real change (SRC) calculated from day 1, and mean changes observed during training camps.

**Camp Details**	**Camp 1**	**Camp 2**	**Camp 3**	**Camp 4**
	Pre-TDU	Early season	Pre-Volta	Pre-TDF
	December 2012	January 2013	March 2013	May 2013
	Sea-level	Sea-level	Sea-level	Altitude
Days	9	10	10	13
n	21	8	7	8
**Body mass (kg)**	68.8 ± 5.9	69.0 ± 6.7	64.5 ± 5.7	67.7 ± 6.1
SWC/SRC	2.6/3.5	2.9/6.5	2.7/6.0	2.7/6.0
Daily change	0.0 ± 0.1	−0.1 ± 0.1	−0.1 ± 0.1	−0.1 ± 0.1
**Skinfolds (sum 7 mm)**	41.8 ± 8.4	43.8 ± 7.1	38.2 ± 4.5	40.6 ± 3.9
SWC/SRC	6.0/5.1	4.8/6.9	3.5/4.7	2.9/3.8
Weekly change	-	−1.9 ± 2.2	-	-
**USG**	1.0194 ± 0.0066	1.0183 ± 0.0072	1.0154 ± 0.0076	1.0138 ± 0.0046
SWC/SRC	0.1954/0.0040	0.2110/0.0070	0.2250/0.0080	0.1371/0.0045
Daily change	−0.0001 ± 0.0009	0.0002 ± 0.0011	0.0007 ± 0.0011	0.0003 ± 0.0006
**Resting HR (bpm)**	-	-	-	44 ± 8
SWC/SRC	-	-	-	6/8
Daily change	-	-	-	0.1 ± 0.3
**SpO_2_ (%)**	-	-	-	93.3 ± 1.3
SWC/SRC	-	-	-	0.4/1.3
Daily change	-	-	-	0.1 ± 0.2
**Sleep duration (h)**	-	-	-	8.4 ± 0.4
SWC/SRC	-	-	-	1.6/0.4
Daily change	-	-	-	0.0 ± 0.0
**Sleep quality (1–5)**	3.6 ± 0.5	4.0 ± 0.5	3.9 ± 0.4	3.8 ± 0.5
SWC/SRC	4.1/0.3	4.0/0.5	2.9/0.4	3.7/0.5
Daily change	0.0 ± 0.1	0.0 ± 0.0	0.0 ± 0.1	0.0 ± 0.1

Data presented as mean ± SD. TDU: Tour Down Under; Volta: Volta a Catalunya; TDF: Tour de France; USG: Urinary specific gravity; HR: Heart rate; SpO_2_: Peripheral oxygen saturation.

**Table 2:** Measurements of elite swimmers on day 1 of training camps, smallest worthwhile change (SWC) and smallest real change (SRC) calculated from day 1, and mean changes observed during training camps.

**Camp Details**	**Camp 1**	**Camp 2**	**Camp 3**	**Camp 4**
	Early-season	Pre-London	Pre-Kazan	Pre-Rio
	January 2012	July 2012	June 2015	May 2016
	Sea-level	Altitude	Altitude	Altitude
Days	19	8	4	18
n	13	12	7	8
**Body mass (kg)**	65.0 ± 5.1 (F)	-	57.3 ± 7.5 (F) 73.1 ± 7.3 (M)	55.4 ± 4.3 (F) 77.2 ± 11.5 (M)
SWC/SRC	2.2/3.6	-	3.0/7.7	6.3/14.1
Daily change	−0.1 ± 0.1	-	−0.2 ± 0.1	−0.1 ± 0.1
**Skinfolds (sum 7 mm)**	67.1 ± 9.9 (F)	79.4 ± 15.6	57.1 ± 12.3 (F) 66.1 ± 20.7 (M)	47.8 ± 5.4 (M)
SWC/SRC	4.4/8.3	5.9/13.0	7.6/16.3	7.3/17.8
Weekly change	-	−8.1 ± 4.1	-	−2.0 ± 0.5
**USG**	1.0150 ± 0.0046	1.0215 ± 0.0054	1.0180 ± 0.0070	1.0172 ± 0.0060
SWC/SRC	0.1352/0.0035	0.1594/0.0043	0.2061/0.0073	0.1774/0.0059
Daily change	-	−0.0003 ± 0.0013	−0.0005 ± 0.0020	0.0001 ± 0.0002
**Resting HR (bpm)**	-	54 ± 7	54 ± 7	53 ± 8
SWC/SRC	-	4/6	4/7	5/8
Daily change	-	0 ± 1	0 ± 2	0 ± 0
**SpO_2_ (%)**	-	95.3 ± 1.7	95.0 ± 1.6	94.0 ± 1.1
SWC/SRC	-	0.5/1.4	0.5/1.7	0.3/1.1
Daily change	-	0.2 ± 0.5	0.0 ± 0.5	0.1 ± 0.0
**Sleep duration (h)**	8.2 ± 0.2	-	-	7.5 ± 0.3
SWC/SRC	0.7/0.2	-	-	1.1/0.3
Daily change	0.0 ± 0.0	-	-	0.0 ± 0.0
**Sleep quality (1–5)**	3.7 ± 0.8	-	-	2.2 ± 0.7
SWC/SRC	6.3/0.6	-	-	9.4/0.7
Daily change	0.0 ± 0.1	-	-	0.1 ± 0.0

Data presented as mean ± SD. USG: Urinary specific gravity; HR: Heart rate; SpO_2_: Peripheral oxygen saturation.

**Table 3:** Percent of athletes flagged against the smallest worthwhile change (SWC) and smallest real change (SRC) calculated from day 1, and z-scores calculated from the daily group change for each measure for all training camps.

Measure of Change	SWC	SRC	z-Score 1.0	z-Score 1.5
Body mass	0 ± 0	0 ± 0	33 ± 5	17 ± 8
USG	0 ± 0	27 ± 18	35 ± 8	18 ± 7
Resting HR	32 ± 16	22 ± 16	32 ± 5	11 ± 8
SpO_2_	63 ± 8	25 ± 13	37 ± 4	16 ± 6
Sleep duration	20 ± 15	60 ± 10	30 ± 2	18 ± 7
Sleep quality	0 ± 0	49 ± 11	36 ± 9	19 ± 8

Data presented as mean ± SD. USG: Urinary specific gravity; HR: Heart rate; SpO_2_: Peripheral oxygen saturation.
